# Investigations on the Phase Transformations, Equilibria and Athermal ω in Ni-Ga-Cr Ternary System

**DOI:** 10.3390/ma15217617

**Published:** 2022-10-30

**Authors:** Jingjing Ruan, Yuyuan Chen, Kosei Kobayashi, Nobufumi Ueshima, Katsunari Oikawa

**Affiliations:** 1 Institute for Advanced Studies in Precision Materials, Yantai University, Yantai 264005, China; 2 Department of Metallurgy, Graduate School of Engineering, Tohoku University, Aramaki-aza Aoba 6-6-02, Aoba-ku, Sendai 980-8579, Japan

**Keywords:** phase diagram, shape memory alloys, omega phase

## Abstract

In the present work, the phase equilibria of the Ni-Ga-Cr ternary system at 850, 1000 and 1150 °C were experimentally investigated to provide the essential data for developing the high-entropy shape memory alloys (HESMAs) containing Ni, Ga and Cr. At 850 °C, in the Ni-rich portion, the B2 phase shows equilibrium with the L12 phase when the Cr content is less than 10.49 at. %, while displaying the equilibrium with L12 and BCC phases when the Cr content increases. The B2 + L12 + BCC changes into B2 + FCC + BCC three-phase equilibria from 850 to 1150 °C, as the L12 phase region becomes narrow with rising temperature. The two-phase equilibrium, B2 + BCC, was found at all the isothermal sections investigated. Other three-phase equilibria were also discovered: B2 + α-Cr3Ga + BCC and Ni2Ga3 + α-Cr3Ga + L at 850 °C, and B2 + α-Cr3Ga + L at 1000 °C. Significantly, an athermal ω intermetallic compound with the space group of P$\bar{3}$m1 was observed distributing at the B2 phase in the quenched Ni45.98-Ga25.50-Cr28.52, Ni42.23-Ga15.70-Cr42.07 and Ni16.54-Ga13.63-Cr69.83 (at. %) alloys after being annealed at 1150 °C for 10 days. The high-resolution transmission electron microscopy (HRTEM) results reveal that the ω shows a crystallographic orientation of $[1\bar{1}0{]}_{B2}//[11\bar{2}0{]}_{\omega }$; ${\left(111\right)}_{B2}//{\left(0001\right)}_{\omega }$ with the B2 parent phase.

## 1. Introduction

In recent years, the materials called high-entropy alloys (HEAs) [1,2] containing five or more principle constituents with or near equiatomic ratio have attracted much attention due to their good properties, including high melting points, good high-temperature strength, attractive ductility, excellent wear resistance, good shape memory effect, etc. [3,4,5,6,7,8,9]. Among the B2-based shape memory alloys, NiAl- and NiGa-based alloys attract much attention. The NiGaCo [7], NiGaFe [8] and NiAlCo [7] systems are known as ferromagnetic shape memory alloys. Moreover, Cr additions increase the martensitic transformation temperature of CoNiGa B2 shape memory alloys [10]. Recently, a CoNiCuAlGaIn high entropy shape memory alloy has been developed successfully using a multi-component approach [9]. The CALPHAD method is a useful computational tool for the design of HEAs as it can provide the necessary information, including equilibrium phases, phase composition, transformation temperature, etc., through the thermodynamic database [11]. Therefore, we have investigated the Ni-Ga-Cr ternary system to provide the essential experimental data for establishing the thermodynamic database for developing the Ni, Ga and Cr incorporated high-entropy shape memory alloys (HESMAs).

**Figure 1:**
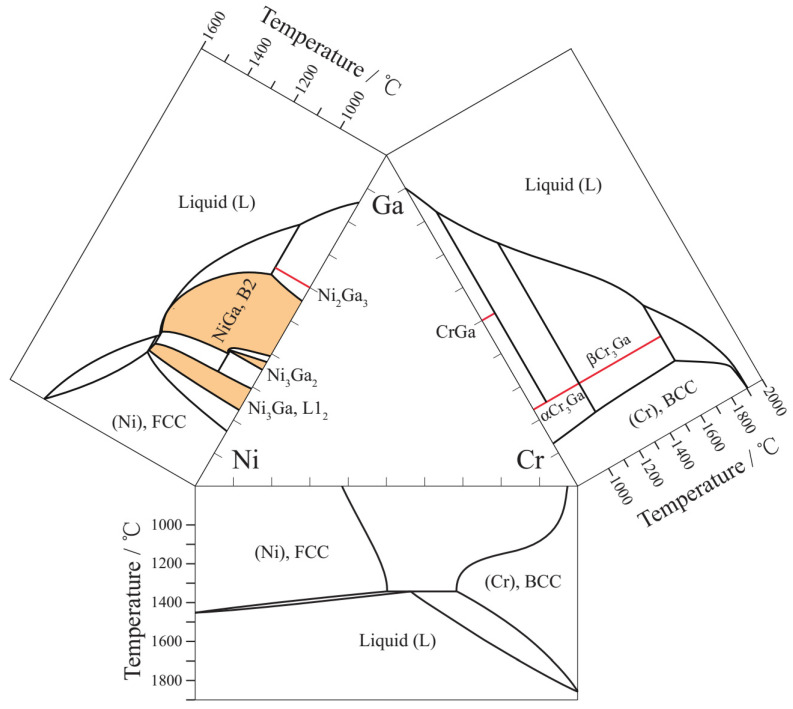
Binary phase diagrams constituting the Ni-Ga-Cr ternary system, and the intermetallic compounds are painted with color.

Figure 1 shows the three binary phase diagrams, Ni-Cr [12], Ni-Ga [13] and Cr-Ga [14] constituting the Ni-Ga-Cr ternary system. Four kinds of intermetallic compounds, Ni3Ga, Ni3Ga2, NiGa and Ni2Ga3, were reported in the Ni-Ga system at elevated temperatures. The Ni3Ga is a L12-ordered phase, while the NiGa is a B2-ordered phase. No intermetallic compound is seen in Ni-Cr system above 600 °C. The CrGa intermetallic compound with B2 structure was reported in the Cr-Ga system, and represented as CrGa, rather than B2 in the present work. Moreover, the Cr3Ga phase was discovered to show an allotropic transformation from β- to α-Cr3Ga, the former is a high-temperature phase, while the other exists at a low-temperature range. To date, only a few studies have been conducted on the Ni-rich portion of the Ni-Ga-Cr ternary system, and the studies are mainly related to the influence of the Cr addition on the Ni3Ga phase, including solubility [15] and lattice misfit [16]. Therefore, we tried to obtain the experimental information for the whole isothermal section of the Ni-Ga-Cr ternary system for the establishment of the database of HESMAs.

## 2. Experimental Details

The alloys weighing 20 g each were prepared from high purity Ni (99.99 wt. %), Cr (99.995 wt. %) and Ga (99.9999 wt. %) by arc-melting under an argon gas atmosphere. The alloy compositions with the atomic percentage are listed in Table 1. The plate-like samples were cut from the ingots and then sealed in the glass capsule filled with argon gas. The heat-treatments for the plate-like samples were performed at 850 °C for 60 days, 1000 °C for 35 days and 1150 °C for 10 days. The annealing time for the alloys containing liquid phase was shortened to 2 h due to the high diffusion rate caused by the presence of liquid phase. Cold rolling was performed on the alloy utilized for determining the phase boundaries of the L12 and Ni-rich FCC phases before heat treatment. After heat treatments, the annealed alloys were quenched in ice water. The annealed samples were polished by a vibrating polishing machine filled with OP-S suspension for 14 h after the pre-polishing by the abrasive paper with different numbers.

Microstructure observations were conducted by field-emission scanning electron microscopy (FE-SEM). Composition analyses were performed by X-ray energy dispersive spectroscopy (EDS) and wavelength dispersive spectroscopy (WDS) equipped on a field-emission electron probe microanalyzer (FE-EPMA). Liquid phases were measured by area analysis of EDS. Ion-milling was introduced to prepare the samples for high-resolution transmission electron microscopy (HRTEM) analyses. The HRTEM and electron back-scattered diffraction (EBSD) equipped FE-SEM were adopted to identify the crystal structure of the precipitates.

## 3. Results and Discussions

### 3.1. Microstructure Analyses and Phase Identifications

**Figure 2:**
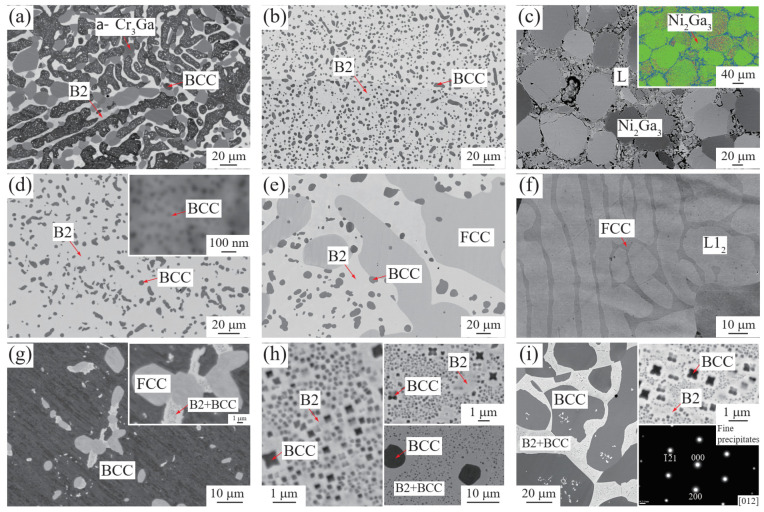
Microstructures of: (**a**) Ni9.24-Ga20.81-Cr69.95 (at. %, 60 days), (**b**) Ni46.31-Ga25.36-28.33Cr (60 days) and (**c**) Ni33.23-Ga62.74-Cr4.03 (2 h, the inset is the EBSD result) annealed at 850 °C, (**d**) Ni44.19-Ga26.80-Cr29.01 (the inset is a high magnification image), (**e**) Ni50.20-Ga21.02-Cr28.78 and (**f**) cold-rolled Ni77.85-Ga18.47-Cr3.68 annealed at 1000 °C for 35 days, (**g**) Ni7.40-Ga2.68-Cr89.92 (the inset is high magnification image), (**h**) Ni45.98-Ga25.50-Cr28.52 (the insets are the microstructures formed when annealed at 1150 °C for 24 h (upper) and 1135 °C for 10 days (lower)) and (**i**) Ni16.54-Ga13.63-Cr69.83 annealed at 1150 °C for 10 days (the insets are the NiGa + BCC region (upper) and the SAD pattern of fine precipitates (lower)).

Figure 2a shows a three-phase equilibrium, B2 + α-Cr3Ga + BCC (Cr), in the Ni9.24-Ga20.81-Cr69.95 (at. %) alloy annealed at 850 °C for 60 days. According to the compositional analyses, the light contrast phase might be B2 phase with Ni and Ga rich, while the phase with dark contrast might be a BCC phase with Cr rich. Figure 2b displays the morphology of the B2 + BCC in the Ni46.31-Ga25.36-Cr28.33 alloy annealed at 850 °C for 60 days. The BCC was found to be spherical in shape and exhibits dark contrast. Figure 2c shows the microstructure of the Liquid (L) + Ni2Ga3 equilibrium obtained in the Ni33.23-Ga62.74-Cr4.03 alloy annealed at 850 °C for 2 h. The coarse precipitates are confirmed to be Ni2Ga3 phase rather than B2 phase, as indicated by the EBSD result in the inset in Figure 2c, where the Ni2Ga3 phase is represented by green color.

Figure 2d shows the microstructure obtained from the Ni44.19-Ga26.80-Cr29.01 alloy annealed at 1000 °C for 35 days, where the phase with dark contrast is the BCC, while the B2 shows a light contrast. Moreover, the very fine BCC precipitates with a spherical shape are noted, as shown in the inset in Figure 2d. It is different from that of the alloy annealed at 850 °C in which no tiny BCC is seen (Figure 2b). The presence of nano-scaled BCC might be precipitated during the quenching of 1000 °C. Three-phase equilibrium, FCC + B2 + BCC, was observed in the Ni50.20-Ga21.02-Cr28.78 alloy annealed at 1000 °C for 35 days, as shown in Figure 2e. The phase with dark contrast is BCC, while the B2 phase shows the light contrast. Figure 2f shows the FCC + L12 (Ni3Ga-rich) microstructure obtained in the Ni77.85-Ga18.47-Cr3.68 alloy after being annealed at 1000 °C for 35 days after cold rolling. The coarsened FCC phases with the elongated shape are noted.

Figure 2g shows the three-phase equilibrium among FCC, B2 and BCC phases in the Ni7.40-Ga2.68-Cr89.92 alloy annealed at 1150 °C for 10 days, where the BCC phase shows dark contrast, and grey FCC and light-contrast B2 phases are embedded in the BCC phase. The nano-scaled cuboidal BCC precipitates, which are thought to precipitate during the quenching process of the annealed sample, are seen to be distributed in the B2 phase, as indicated in the inset in Figure 2g. It means the precipitation of BCC in the B2 phase is too fast to be yielded by ice water quenching. The microstructure constituted of B2 and BCC phases, where the BCC exhibits a cuboidal morphology, is observed in the Ni45.98-Ga25.50-Cr28.52 alloy annealed at 1150 °C for 10 days, as shown in Figure 2h. Interestingly, a similar microstructure was also observed in the alloy with the same composition when the annealing time was decreased to 24 h, as shown in the upper inset in Figure 2h. It is seen that the size of the cuboidal BCC phase changes very little between the short- (24 h) and long-term (10 days) annealed alloys. Additionally, the lower inset in Figure 2h shows the microstructure obtained from the Ni45.98-Ga25.50-Cr28.52 alloy annealed at 1135 °C for 10 days, besides the large-scaled BCC phases, nano-scaled cuboidal BCC precipitates are noted. Therefore, the cuboidal BCC phases seen in Figure 2h and its inset are believed to be precipitated during the quenching process, and the Ni45.98-Ga25.50-Cr28.52 alloy is believed to be a single B2 phase when annealed at 1150 °C. Figure 2i displays the B2 + BCC equilibrium in the Ni16.54-Ga13.63-Cr69.83 alloy annealed at 1150 °C for 10 days. The coarse phase with dark contrast is the BCC phase. Moreover, the tiny cuboidal precipitates (as seen in the upper inset in Figure 2i) embedded in the light contrast B2 phase were confirmed to be BCC, as indicated by the SADP from [012] direction of the tiny precipitates (the bottom inset in Figure 2i).

**Figure 3:**
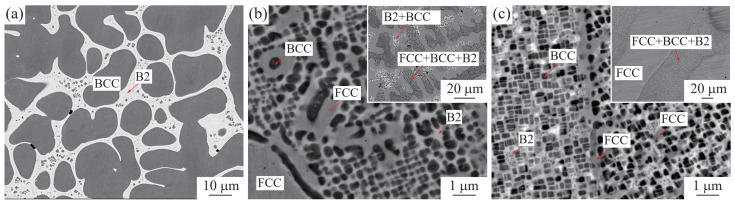
Microstructures of (**a**) the as-cast Ni16.54-Ga13.63-Cr69.83 alloy, (**b**) as-cast and (**c**) annealed Ni42.23-Ga15.70-Cr42.07 alloys (the insets are the images with low magnification). Annealing is performed at 1150 °C for 10 days for Ni42.23-Ga15.70-Cr42.07 (at. %) alloys.

Figure 3a shows the microstructure of the cast Ni16.54-Ga13.63-Cr69.83 alloy, and the BCC is suggested to precipitate from the molten alloy during the solidification first, then, the remaining liquid reacts with the precipitated BCC to form the B2 phase through a peritectic reaction. Moreover, during the cooling of the cast alloy, the tiny BCC phase starts to precipitate from the B2 phase, as suggested in Figure 2g,h, that the precipitation of BCC in B2 is very fast. Meanwhile, not like that in the annealed Ni16.54-Ga13.63-Cr69.83 alloy where the tiny B2 phase is seen embedded in the large BCC phase (Figure 2i), no tiny B2 phase is seen in the large BCC phase in cast alloy (Figure 3a). Therefore, the tiny B2 phase seen in the annealed Ni16.54-Ga13.63-Cr69.83 alloy is thought to form during the annealing process. Figure 3b,c show the microstructures obtained from the Ni42.23-Ga15.70-Cr42.07 alloy under as-cast and after annealing at 1150 °C for 10 days. In the as-cast condition, the dendrite-like FCC, B2 + BCC and FCC + BCC + B2 regions are observed, as seen in the inset in Figure 3b. After annealing, the microstructure consists of large FCC and FCC + BCC + B2 regions, as seen in the inset in Figure 3c. In the FCC + BCC + B2 region (Figure 3c), two kinds of BCC are observed, a large one and a small one. The cuboidal BCC, which shows an edge length close to 500 nm, is believed to form during the annealing process as the mean composition of this alloy is located at the three-phase region of FCC + BCC + B2 at 1150 °C, as indicated by Figure 2g. The very tiny BCC phase is found to be distributed on the B2 phase and is thought to be precipitated during the ice water quenching process. It is the same as that shown in Figure 2g–I, where the tiny BCC phase is seen precipitated in the B2 phase. The co-existing FCC in the B2 phase in the matrix indicates the improved ductility of this alloy. Furthermore, the observed cuboidal BCC was discovered to coherently precipitate on the B2 matrix with cuboidal morphology.

### 3.2. Observations of the ω Phase

In the present work, an athermal ω phase was observed in the quenched Ni45.98-Ga25.50-Cr28.52, Ni42.23-Ga15.70-Cr42.07 and Ni16.54-Ga13.63-Cr69.83 alloys after annealing at 1150 °C for 10 days. The details related to the former two alloys will be presented in this part. It is now believed that the ordering of the ω phase is inherently derived from its parent phase [17,18,19,20,21,22,23,24]. For the ordered B2 parent, the ω phase becomes inherently ordered and is classified into hexagonal (P63/mmc) and trigonal (P$\bar{3}$m1) crystal structures. The P63/mmc is formed during the aging process, while the P$\bar{3}$m1 is usually found in the as-quenched alloys.

**Figure 4:**
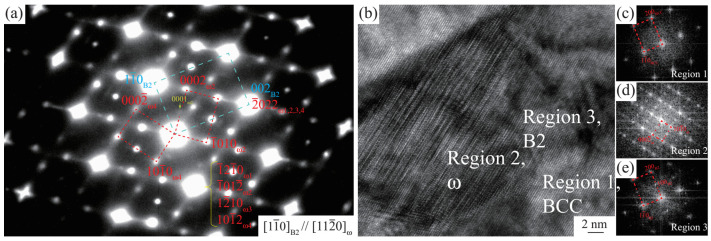
(**a**) Selected-area diffraction (SAD) pattern obtained from the matrix in the quenched Ni45.98-Ga25.50-Cr28.52 alloy after annealing at 1150 °C for 10 days. (**b**) High-resolution transmission electron microscopy (HRTEM) image obtained from the quenched Ni42.23-Ga15.70-Cr42.07 alloy after annealing at 1150 °C for 10 days. (**c**–**e**) Fast Fourier Transforms (FFTs) of regions 1, 2 and 3 in (**b**).

The diffuse streaking is seen in the selected-area diffraction (SAD) pattern obtained from the matrix in the quenched Ni45.98-Ga25.50-Cr28.52 alloy after being annealed at 1150 °C for 10 days, as shown in Figure 4a, and is a typical phenomenon of the presence of a diffuse ω structure. Four ω variants are noted and found to be in the space group of P$\bar{3}$m1 due to the presence of ${\left(0001\right)}_{\omega }$ diffraction (the very weak spot located at $\frac{1}{3}{\left(1\bar{1}1\right)}_{B2}$ position in Figure 4a), which is forbidden for the P63/mmc space group. The observed ω phase shows a crystallographic orientation of ${\left[1\bar{1}0\right]}_{B2}//{\left[11\bar{2}0\right]}_{\omega }$; ${\left(111\right)}_{B2}//{\left(0001\right)}_{\omega }$ with parent phase B2. Moreover, it is reasonable to understand the appearance of P$\bar{3}$m1 rather than the P63/mmc space group as the ice water quenching was performed after the annealing of the Ni45.98-Ga25.50-Cr28.52 alloy at 1150 °C. Figure 4b shows the HRTEM result obtained from the FCC + BCC + B2 region (Figure 3c) in the quenched Ni42.23-Ga15.70-Cr42.07 alloy after annealing at 1150 °C for 10 days. No FCC is identified due to the very small region observed. The BCC phase, ω with P$\bar{3}$m1 space group, and the remaining B2 phases are identified, as suggested in Figure 4c–e obtained, respectively, from the regions 1–3 in Figure 4b using the Fast Fourier Transform (FFT) method.

### 3.3. Determination of the Phase Boundary

**Figure 5:**
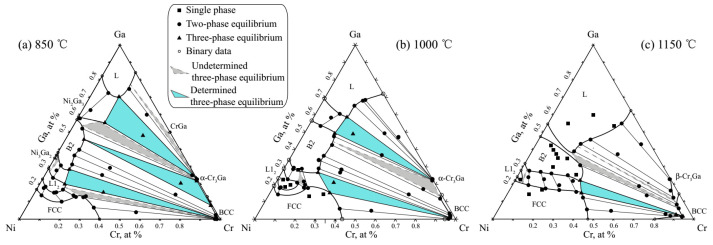
(**a**–**c**). Constructed isothermal sections of the Ni-Ga-Cr ternary system at 850 (**a**), 1000 (**b**) and 1150 °C (**c**).

All the compositions measured for the phases in the annealed alloys using EPMA and HRTEM are listed in Table 1, where Cr is selected as the balance element, and the constructed three isothermal sections of the Ni-Ga-Cr ternary system at 850, 1000 and 1150 °C are shown in Figure 5a–c. The three-phase regions are represented by triangles.

At 850 °C, as seen in Figure 5a, seven three-phase equilibrium regions are indicated. The CrGa + L + α-Cr3Ga, L12 + Ni3Ga2 + B2 and FCC + L12 + BCC regions are suggested to be very small. The solubility of Cr in L12 was measured as 13.17 at. %. The occupation of Cr in the L12 phase changes from Ga to Ni sites with the increasing Cr content. Moreover, the solubility of Ga in the FCC phase decreases first and then increases with increasing Cr content. The Ni shows very little solubility in the α-Cr3Ga intermetallic compound. Four three-phase regions, B2 + L + α-Cr3Ga, B2 + BCC + α-Cr3Ga, B2 + L12 + FCC and B2 + BCC + FCC were found at 1000 °C, as displayed in Figure 5b. The solubility of Cr in L12 decreases to 10.53 at. % compared with that at 850 °C. The B2 phase shows a larger solubility to Cr compared with that at 850 °C. The Ga solubility in the FCC phase changes with increasing Cr content and shows a similar trend as that which acts at 850 °C. Only the B2 + FCC + BCC three-phase region is observed due to the very narrow range of the other three three-phase regions (L + β-Cr3Ga + BCC, B2 + L + BCC and B2 + FCC + L12) at 1150 °C, as shown in Figure 5c. The solubility of Cr in B2 is increased compared with that in the lower temperatures (850 and 1000 °C), and found to be about 32.44 at. %. The solubility of Ni in Cr3Ga increases when the allotropic transformation from α- to β-Cr3Ga has happened.

## 4. Conclusions

In the present work, the Ni-Ga-Cr ternary system was investigated experimentally by FE-EPMA, HRTEM, FE-SEM and EBSD. Three isothermal sections at 850, 1000 and 1150 were determined. The details are as follows:

(1) A trigonal intermetallic compound ω with the space group of P$\bar{3}$m1 was observed in the matrix in the water-quenched Ni45.98-Ga25.50-Cr28.52, Ni42.23-Ga15.70-Cr42.07 and Ni16.54-Ga13.63-Cr69.83 alloys after annealing at 1150 °C for 10 days, and shows a crystallographic orientation of $[1\bar{1}0{]}_{B2}//[11\bar{2}0{]}_{\omega }$; ${\left(111\right)}_{B2}//{\left(0001\right)}_{\omega }$ with the B2 parent phase.

(2) The precipitation of BCC on the B2 phase was found to be very fast and cannot be suppressed by ice water quenching. In the Ni42.23-Ga15.70-Cr42.07 alloy annealed at 1150 °C for 10 days, the FCC was found to co-exist with the B2 phase in the matrix, thus the improved ductility of this alloy is suggested. Moreover, the observed cuboidal BCC was discovered to coherently precipitate on the B2 matrix with cuboidal morphology in this alloy.

(3) The solubility of Cr in B2 was found to increase with the rising temperature. The solubility of the Ni in the Cr3Ga phase increases when the allotropic transformation from α- to β-Cr3Ga has happened. The solubility of Cr in the L12 phase decreases from ~13.17 to 10.53 at. % when the temperature rises from 850 to 1000 °C.

(4) Three-phase equilibria, L12 + B2 + BCC, B2 + α-Cr3Ga + BCC and Ni2Ga3 + α-Cr3Ga + L, were discovered at 850 °C. At 1000 °C, the three-phase equilibria close to the Ni-rich region become L12 + B2 + FCC and B2 + FCC + BCC. The B2 + FCC + BCC three-phase equilibrium was discovered at 1150 °C.

## Figures and Tables

**Table 1:** The measured chemical compositions of the equilibrium phase in the annealed alloys.

T/°C	Alloys/at. % (EDS) Cr Balance	Equilibrium Phase (WDS)			Composition/at. %					
		Phase 1	Phase 2	Phase 3	Phase 1		Phase 2		Phase 3	
					Ni	Ga	Ni	Ga	Ni	Ga
850	Ni1.34-Ga25.24	B2	α-Cr_3_Ga		42.66	52.1	0.67	22.46		
	Ni15.19-Ga14.09	B2	BCC		50.27	39.89	1.12	2.27		
	Ni50.48-Ga15.50	B2	L1_2_	BCC	60.8	28.71	67.44	20.12	1.54	0.19
	Ni67.61-Ga27.87	L1_2_	B2		71.49	23.64	63.98	30.78		
	Ni14.47-Ga48.11	L	α-Cr_3_Ga	Ni_2_Ga_3_	15.13	70.53	0.05	23.37	29.81	55.44
	Ni39.91-Ga4.0	BCC	FCC		1.81	0.03	59.66	6.6		
	Ni9.24-Ga20.81	BCC	α-Cr_3_Ga	B2	0.68	7.98	0.59	22.05	44.88	45.95
	Ni40.02-Ga29.62	BCC	B2		1.57	0.93	54.32	36.6		
	Ni66.27-Ga13.64	L1_2_	FCC		69.86	16.97	65.5	12.75		
	Ni78.94-Ga18.02	FCC	L1_2_		82.13	13.63	77.84	18.44		
	Ni60.30-Ga13.21	FCC	BCC		61.87	12.63	2.65	0.1		
	Ni73.75-Ga15.99	FCC	L1_2_		73.89	10.04	75.13	15.21		
	Ni33.23-Ga62.74	Ni_2_Ga_3_	L		41.38	57.49	19.46	72.56		
	Ni6.52-Ga60.09	α-Cr_3_Ga	L		0	22.95	7.89	74.9		
	Ni46.31-Ga25.36	B2	BCC		58.03	31.44	1.94	0.45		
1000	Ni18.11-Ga37.27	α-Cr_3_Ga	B2		0.51	22.21	36.78	47.61		
	Ni16.16-Ga14.37	BCC	B2		1.38	3.23	47.46	36.44		
	Ni50.20-Ga21.02	BCC	FCC	B2	2.38	0.65	55.11	15.09	55.07	26.91
	Ni66.65-Ga28.55	L1_2_	B2		72.22	22.84	65.91	29.34		
	Ni10.70-Ga49.40	L	α-Cr_3_Ga		17.76	65.28	0.08	22.88		
	Ni39.61-Ga4.82	BCC	FCC		3.43	0.12	54.74	5.98		
	Ni7.46-Ga17.28	BCC	B2		1.81	11.76	40.22	42.6		
	Ni42.63-Ga28.48	BCC	B2		1.65	1.94	51.15	34		
	Ni4.82-Ga47.26	L	α-Cr_3_Ga		10.67	68.6	0.11	22.8		
	Ni66.37-Ga13.04	FCC			66.37	13.04				
	Ni74.57-Ga22.70	L1_2_			74.57	22.7				
	Ni77.85-Ga18.47	FCC	L1_2_		81.24	14.27	78.71	17.89		
	Ni58.65-Ga14.28	FCC			58.65	14.28				
	Ni44.19-Ga26.80	BCC	B2		2.2	0.89	53.89	29.13		
	Ni32.70-Ga58.53	L	B2		27.41	60.59	40.54	53.51		
	Ni75.75-Ga18.44	L1_2_			75.75	18.44				
	Ni72.52-Ga19.79	L1_2_			72.52	19.79				
	Ni65.0-Ga20.0	L1_2_	FCC		68.45	21.02	66.52	18.08		
	Ni65.0-Ga21.60	FCC	B2		64.64	17.74	62.04	29.27		
	Ni26.55-Ga49.10	L	α-Cr_3_Ga	B2	24.66	58.9	0.25	22.55	35.33	49.51
1150	Ni19.16-Ga37.33	β-Cr_3_Ga	L		3.21	16.37	28.48	45.74		
	Ni16.54-Ga13.63	BCC	B2 including tiny BCC (EDS)		4.31	6.14	41.03	30.96		
	Ni49.14-Ga21.94	B2 including tiny BCC (EDS)	FCC		50.02	25.3	50.62	15.65		
	Ni70.30-Ga24.02	B2	FCC		67.93	27.82	72.57	19.42		
	Ni12.87-Ga53.65	L			12.87	53.65	0	0		
	Ni38.75-Ga4.69	BCC	FCC		10.68	1.8	50.34	5.54		
	Ni12.17-Ga20.77	β-Cr_3_Ga	L		3.83	12.43	34.6	43.26		
	Ni4.63-Ga43.05	L	β-Cr_3_Ga		8.3	55.05	0.51	22.91		
	Ni74.92-Ga22.46	L1_2_			74.92	22.46				
	Ni42.23-Ga15.70	FCC	B2	BCC	48.66	14.8	Not determined	Not determined	Not determined	Not determined
	Ni17.69-Ga18.96	BCC	L		4.23	8.33	40.04	38.98		
	Ni74.93-Ga14.21	FCC			74.93	14.21				
	Ni67.03-Ga16.88	FCC			67.03	16.88				
	Ni45.98-Ga25.50	B2 including tiny BCC (EDS)			45.98	25.5				
	Ni57.25-Ga27.13	B2 including tiny BCC			57.25	27.13				
	Ni51.96-Ga32.25	B2 including tiny BCC			51.96	32.25				
	Ni63.03-Ga22.96	FCC	B2		64.94	17.58	62.6	27.18		
	Ni57.26-Ga19.50	FCC	B2		58.59	16.68	57.72	26.2		
	Ni7.40-Ga2.68	FCC	B2 including tiny BCC (EDS)	BCC	48.34	15.06	45.42	22.14	4.5	1.71
	Ni12.60-Ga2.81	FCC	BCC		48.93	12.36	5.21	1.26		
	Ni49.74-Ga37.35	B2			49.74	37.35				
	Ni49.89-Ga42.38	B2			49.89	42.38				
	Ni49.97-Ga39.84	B2			49.97	39.84				
	Ni45.52-Ga34.92	B2 including tiny BCC (EDS)			45.52	34.92				

## References

[1] Yeh J.W., Chen S.K., Lin S.J., Gan J.Y., Chin T.S., Shun T.T., Tsau C.H., Chang S.Y. (2004). Nanostructured high-entropy alloys with multiple principal elements: Novel alloy design concepts and outcomes. Adv. Eng. Mater..

[2] Cantor B., Chang I.T.H., Knight P., Vincent A.J.B. (2004). Microstructural development in equiatomic multicomponent alloys. Mat. Sci. Eng. A Struct..

[3] Yang T., Zhao Y.L., Tong Y., Jiao Z.B., Wei J., Cai J.X., Han X.D., Chen D., Hu A., Kai J.J. (2018). Multicomponent intermetallic nanoparticles and superb mechanical behaviors of complex alloys. Science.

[4] Miracle D.B., Senkov O.N. (2017). A critical review of high entropy alloys and related concepts. Acta Mater..

[5] Ye Y.F., Wang Q., Lu J., Liu C.T., Yang Y. (2016). High-entropy alloy: Challenges and prospects. Mater. Today.

[6] Chen J., Zhou X.Y., Wang W.L., Liu B., Lv Y.K., Yang W., Xu D.P., Liu Y. (2018). A review on fundamental of high entropy alloys with promising high–temperature properties. J. Alloy. Compd..

[7] Oikawa K., Ota T., Gejima F., Ohmori T., Kainuma R., Ishida K. (2001). Phase equilibria and phase transformations in new B2-type ferromagnetic shape memory alloys of Co-Ni-Ga and Co-Ni-Al systems. Mater. Trans..

[8] Oikawa K., Ota T., Ohmori T., Tanaka Y., Morito H., Fujita A., Kainuma R., Fukamichi K., Ishida K. (2002). Magnetic and martensitic phase transitions in ferromagnetic Ni-Ga-Fe shape memory alloys. Appl. Phys. Lett..

[9] Gerstein G., Firstov G.S., Kosorukova T.A., Koval Y.N., Maier H.J. (2018). Development of B2 shape memory intermetallics beyond NiAl, CoNiAl and CoNiGa. Shape Mem. Superelast..

[10] Decker P., Fortmann J., Salomon S., Krooß P., Niendorf T., Ludwig A. (2019). Influence of Cr alloying (1.5 to 5 at.%) on martensitic phase transformation temperatures in Co-Ni-Ga-Cr thin films. Shape Mem. Superelast..

[11] Andersson J.O., Helander T., Höglund L., Shi P., Sundman B. (2002). Thermo-Calc & DICTRA, Computational Tools For Materials Science. Calphad.

[12] Nash P. (1991). Phase Diagrams of Binary Nickel Alloys.

[13] Okamoto H. (2010). Ga-Ni (Gallium-Nickel). J. Phase Equilib. Diff..

[14] Belgacem-Bouzida A., Djaballah Y., Notin M. (2005). Calorimetric measurement of the intermetallic compounds Cr3Ga and CrGa4 and thermodynamic assessment of the (Cr-Ga) system. J. Alloy. Compd..

[15] Ochial S., Oya Y., Suzuki T. (1984). Alloying behaviour of Ni3Al, Ni3Ga, Ni3Si and Ni3Ge. Acta Metall..

[16] Mishima Y., Ochiai S., Suzuki T. (1985). Lattice parameters of Ni (γ), Ni3Al (γ’) and Ni3Ga (γ’) solid solutions with additions of transition and B-subgroup elements. Acta Metall..

[17] Shao G., Miodownik A.P., Tsakiropoulos P. (1995). ω-phase formation in V-Al and Ti-Al-V alloys. Philos. Mag. A.

[18] Sikka S.K., Vohra Y.K., Chidambaram R. (1982). Omega phase in materials. Prog. Mater. Sci..

[19] Okunishi E., Kawai T., Mitsuhara M., Farjami S., Itakura M., Hara T., Nishida M. (2013). HAADF-STEM studies of athermal and isothermal ω-phases in β-Zr alloy. J. Alloy. Compd..

[20] Sass S.L. (1972). The structure and decomposition of Zr and Ti bcc solid solutions. J. Less-Common Met..

[21] Strychor R., Williams J.C., Soffa W.A. (1988). Phase transformations and modulated microstructures in Ti-Al-Nb alloys. Metall. Trans. A.

[22] Shao G., Tsakiropoulos P. (2000). On the ω phase formation in Cr-Al and Ti-Al-Cr alloys. Acta Mater..

[23] Bendersky L.A., Boettinger W.J., Burton B.P., Biancaniello F.S., Shoemaker C.B. (1990). The formation of ordered ω-related phases in alloys of composition Ti4Al3Nb. Acta Metall. Mater..

[24] Dawson C.W., Sass S.L. (1970). The as-quenched form of the omega phase in Zr-Nb alloys. Metall. Trans..

